# Tibial osteitis caused by Mycobacterium tuberculosis

**DOI:** 10.1099/acmi.0.000960.v4

**Published:** 2025-07-17

**Authors:** Marwa Benjelloun, Naji Tijani, Yassine BenLahlou, Elmostafa Benaissa, Mariama Chadli

**Affiliations:** 1 Microbiology Laboratory, Faculty of Medicine and Pharmacy, Mohammed V Military Training Hospital, Rabat, Morocco; 2 Traumatology Department, Faculty of Medicine and Pharmacy, Mohammed V Military Training Hospital, Rabat, Morocco

**Keywords:** PCR, tibia, tuberculosis

## Abstract

Tuberculosis is a major scourge, posing a serious public health problem in countries where it is endemic. Osteoarticular involvement accounts for 3–5% of all tuberculosis cases and 10–15% of extrapulmonary tuberculosis cases. We report a case of tibial osteitis caused by *Mycobacterium tuberculosis* in a 52-year-old female patient who presented to the trauma department at the Mohammed V Military Teaching Hospital with a painful swelling of the lower part of her left leg. Standard X-rays and computed tomography scans revealed bone involvement, specifically in the tibia. Additional investigations revealed pulmonary consolidation and splenic nodules. Microscopy (Ziehl–Neelsen staining), GeneXpert MTB/RIF and histopathological examination all returned positive results for *M. tuberculosis*. In an endemic context, any persistent and atypical bone lesion should raise suspicion of osteoarticular tuberculosis to enable rapid diagnosis and appropriate therapeutic management. In the absence of malignant tumours and other differential diagnoses, the diagnosis of skeletal tuberculosis must be considered, even in the absence of specific clinical signs.

## Data Summary

No data were reused or generated in this study.

## Introduction

Tuberculosis remains a major public health challenge, particularly in countries where it is endemic. In Morocco, the incidence of tuberculosis was estimated at 94 cases per 100,000 inhabitants in 2022, with extrapulmonary forms accounting for around 46% of notified cases, according to the Moroccan Ministry of Health. Although pulmonary tuberculosis is the most common form, osteoarticular tuberculosis constitutes an important subset of extrapulmonary tuberculosis, representing ~3–5% of all tuberculosis cases and 10–15% of extrapulmonary cases globally [1].

It is characterized by infection of bones, joints, synovial membranes or the spine and can lead to progressive bone destruction if not diagnosed early. The clinical presentation is often nonspecific, sometimes mimicking severe conditions such as malignant tumours [2]. Osteoarticular tuberculosis of the tibia, also known as tibial tuberculous osteitis, is a localized infection caused by the Koch bacillus (*Mycobacterium tuberculosis*). It typically presents as persistent, insidious pain in the tibial region, sometimes accompanied by swelling and systemic symptoms such as fever, fatigue or weight loss – though these are frequently absent in chronic cases. Imaging may show characteristic bone lesions such as osteolytic cavities or sequestra. Diagnosis is confirmed by bone biopsy and detection of *M. tuberculosis* using molecular techniques [3].

We report here the case of a 52-year-old woman with tuberculous involvement of the lower part of the left tibia, presenting as a bone tumour, an uncommon and atypical manifestation with an estimated frequency below 1% of all skeletal tuberculosis cases [3].

## Case report

This case involves a 52-year-old woman with no notable medical history and no known tuberculosis contact. She was admitted for evaluation of a hard, painful swelling on her left lower leg that had been progressively increasing over a year, with no history of trauma. The pain was tingling in nature, more intense at night and worsened by movement. It was initially relieved by analgesics, prompting her to consult a rheumatologist who prescribed non-steroidal anti-inflammatory drugs without clinical improvement. Due to persistent symptoms, she was referred to the trauma department.

On admission, the patient was in good general condition and reported no systemic symptoms such as fatigue, fever, night sweats or weight loss.

Clinical examination revealed a hard, tender mass localized over the distal third of the left tibia. The swelling extended to the ankle but showed no local inflammatory signs such as erythema, warmth or tenderness. The mass was measured on imaging and estimated to be ~6×3 cm.

The differential diagnoses initially included neoplastic pathology and non-specific infections. However, the absence of typical malignant features on imaging – such as periosteal elevation, cortical bone destruction or soft tissue invasion – along with the presence of characteristic lesions (osteolytic aspect with a flame-like periosteal reaction and synovial thickening) supported the hypothesis of osteoarticular tuberculosis.

Pulmonary auscultation was normal, with no crackles or wheezing. Oxygen saturation was 94% on room air. There were no signs of respiratory distress. Cardiovascular examination showed a blood pressure of 120/50 mmHg, a heart rate of 74 bpm and normal heart sounds.

Neurological evaluation was unremarkable (GCS 15/15). She was well-oriented in time and space and exhibited no sensory or motor deficits. Pupils were equal and reactive.

Routine blood tests were mostly within normal limits. The only notable abnormality was an elevated C-reactive protein at 24.2 mg l^−1^. Liver and kidney function were preserved. Serologies for hepatitis B virus, hepatitis C virus and human immunodeficiency virus were negative. Autoimmune markers (rheumatoid factor and antinuclear antibodies) were also negative.

**Fig. 1.**
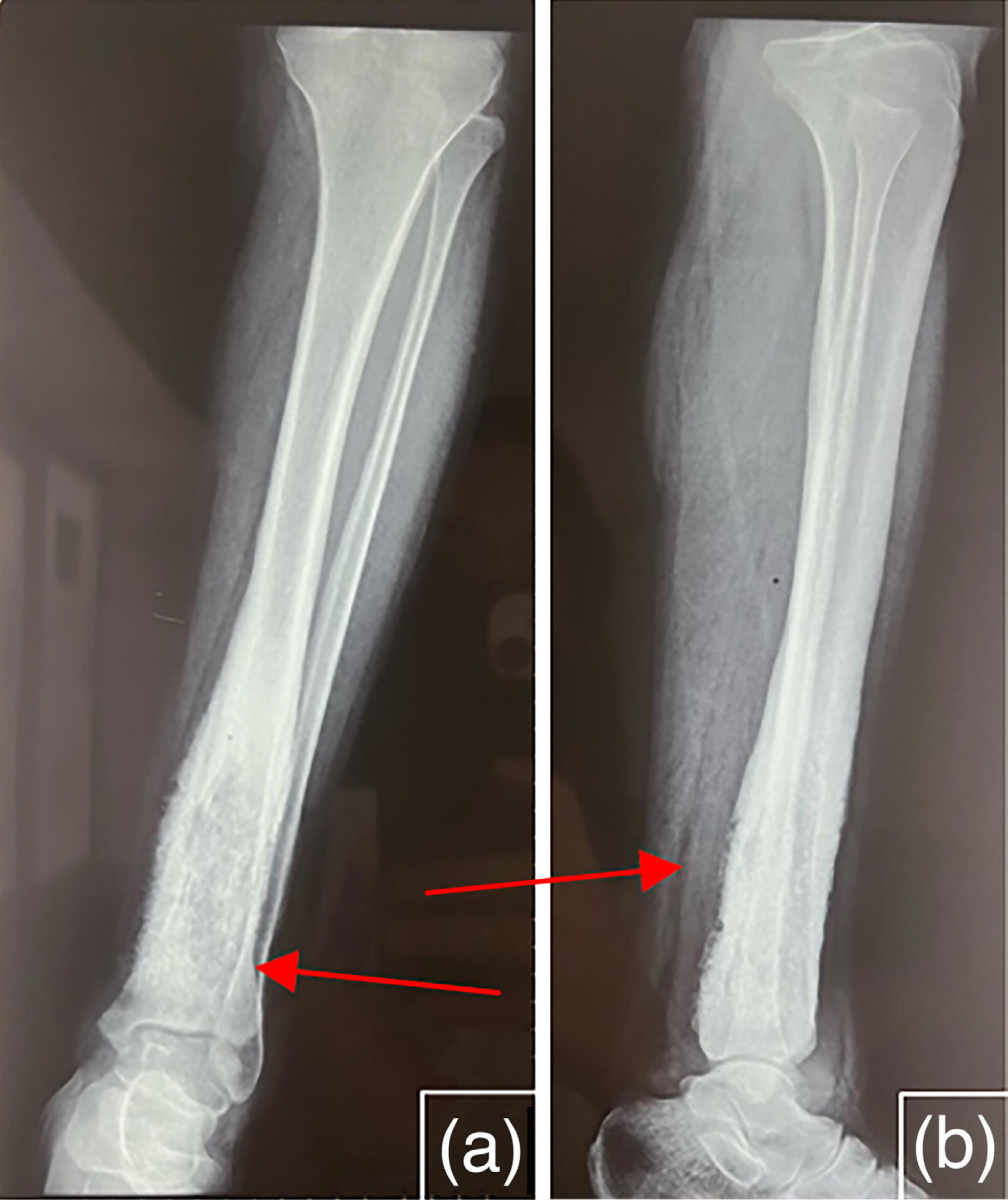
Standard X-ray of the left leg, frontal (a) and lateral (b) views, showing a marked osteosclerosis (indicated by the red arrow) in the distal tibial region.

Standard radiographs of the left leg (AP and lateral views) showed a mixed lytic–sclerotic lesion in the lower diaphyseal–metaphyseal region of the tibia, associated with a ‘flame-like’ periosteal reaction and mild osteosclerosis of the first distal phalanx, without cortical breach (Fig. 1).

**Fig. 2.**
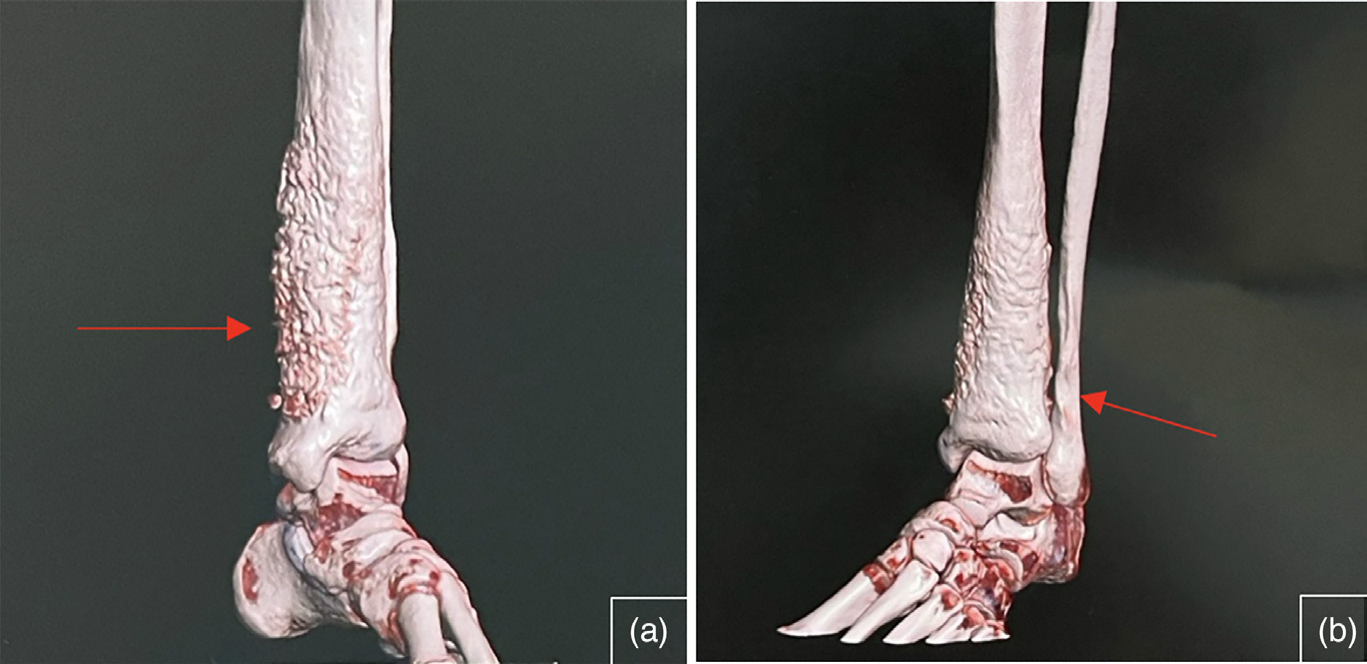
Volumetric reconstruction on CT scan of the left leg showing the synovial thickening (red arrows).

**Fig. 3.**
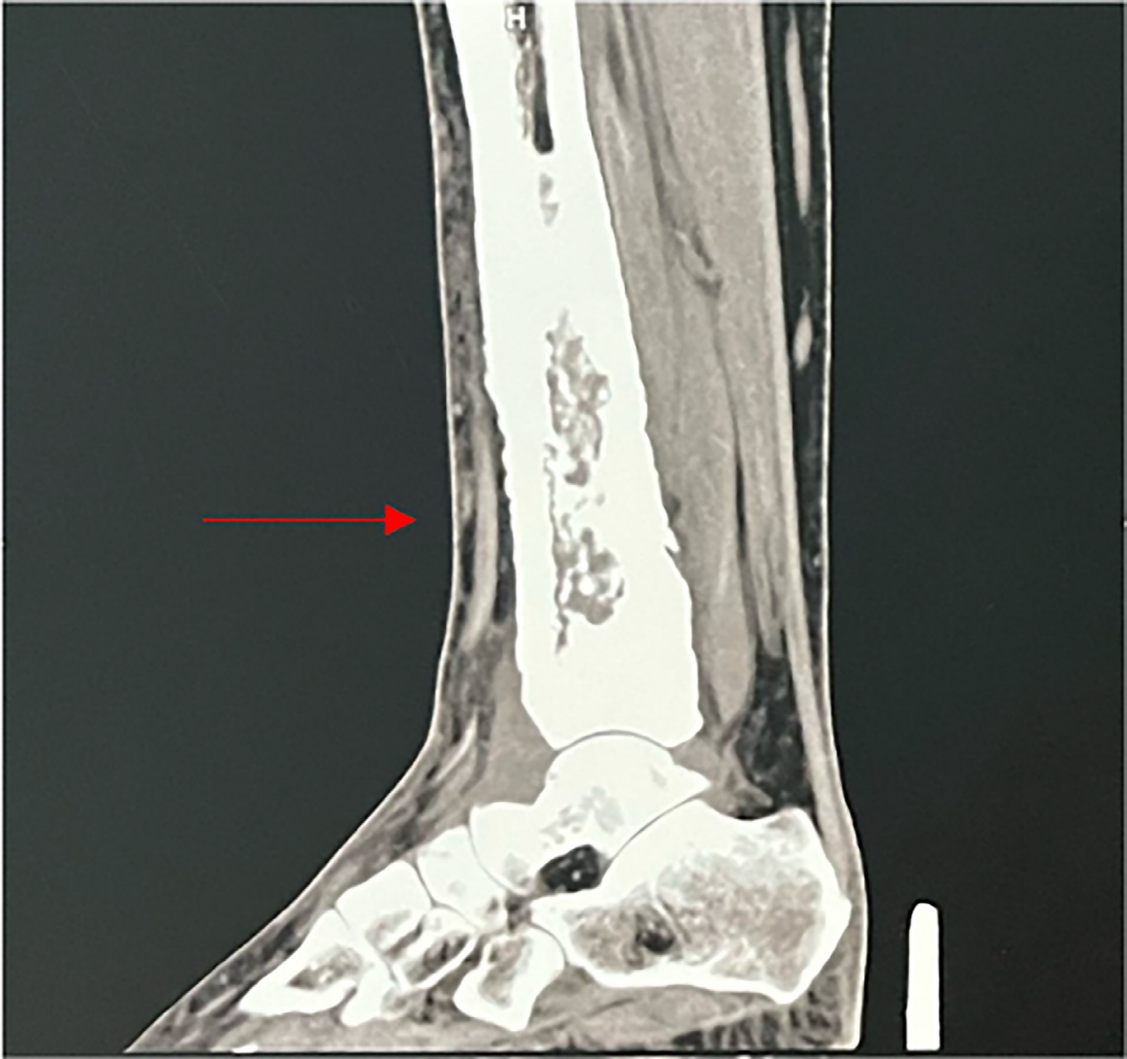
Volumetric reconstructions on CT scan of the left leg with synovial thickening (red arrows).

Computed tomography (CT) and magnetic resonance imaging (MRI) scans revealed an effusion of the talocrural joint and diffuse synovial thickening enhanced by contrast injection (Figs 2 and 3).

Thoracoabdominal-pelvic CT was performed to identify a primary focus and showed a bronchiectasis with signs of superinfection and multiple splenic nodules suggestive of secondary tuberculous involvement.

A bone biopsy was performed by the trauma team for histological and microbiological confirmation. The biopsy confirmed the diagnosis by detecting *M. tuberculosis* through molecular techniques (GeneXpert MTB/RIF), culture and histopathology, guiding appropriate anti-tuberculosis therapy.

## Results

**Fig. 4.**
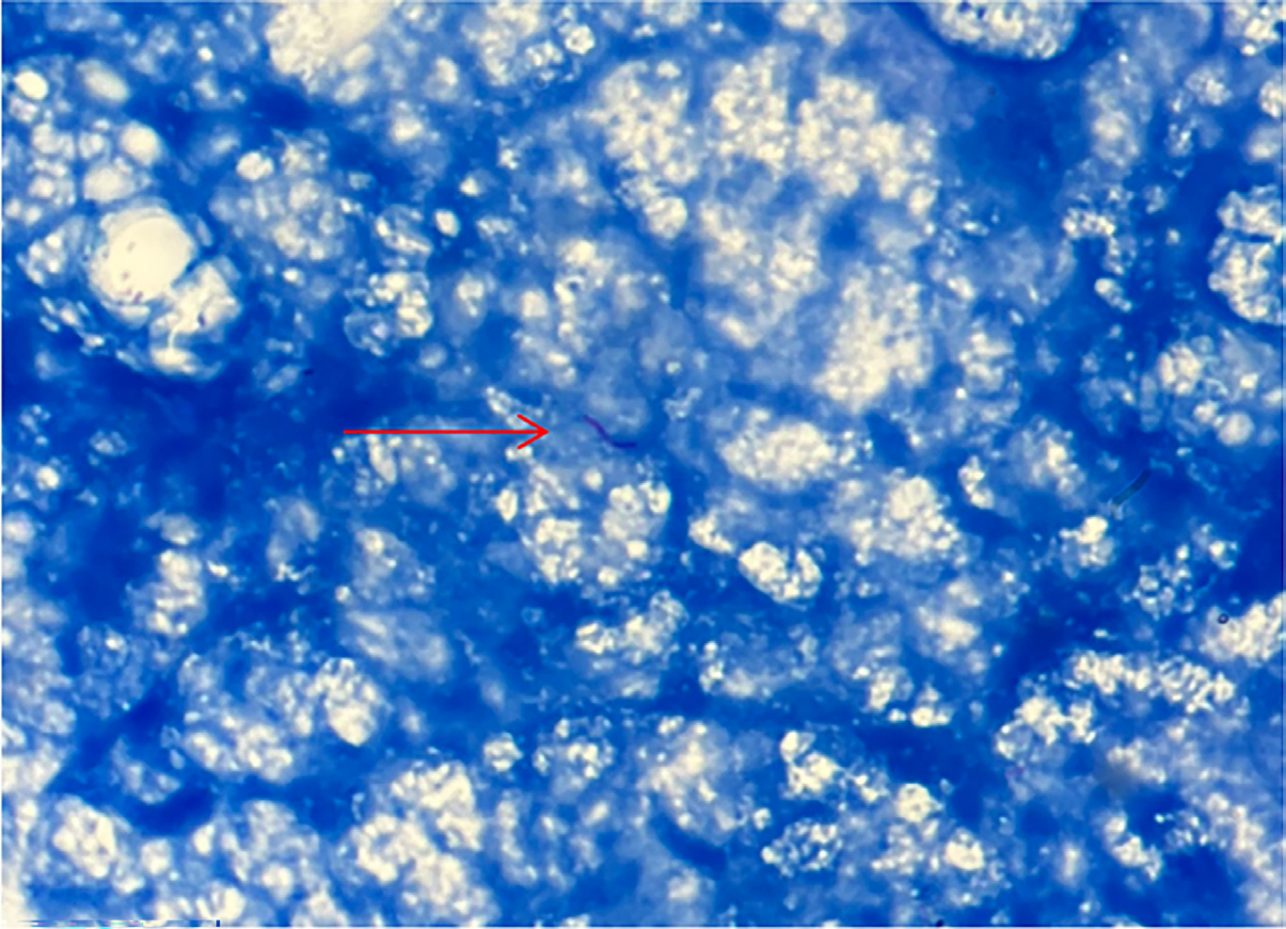
Ziehl–Neelsen staining of bone biopsy specimen. Microscopic view with suspected AFB indicated by red arrow (×1,000). While the image lacks the typical red/purple coloration of AFB due to probable suboptimal staining, the diagnosis was confirmed by molecular, culture and histological methods.

Bacteriological examination of the bone biopsy revealed the presence of acid-fast bacilli (AFB) on Ziehl–Neelsen staining (1–10 AFB per 100 fields), which is highly suggestive of infection with *M. tuberculosis* (Fig. 4). Confirmation was obtained through culture and molecular analysis. Cultures became positive on day 21 in Löwenstein-Jensen medium and on day 14 in liquid Mycobacteria Growth Indicator Tube medium.

Real-time PCR using the GeneXpert MTB/RIF^®^ assay (Cepheid) detected *M. tuberculosis* complex DNA at a low level. No rifampicin resistance-associated mutations were identified, allowing for standard first-line therapy.

Histopathological analysis of the bone tissue revealed caseating granulomas, a hallmark of tuberculosis, surrounded by a dense granulomatous infiltrate composed of lymphocytes and macrophages. This histological picture was consistent with chronic osteitis due to *M. tuberculosis*.

These combined clinical, radiological, microbiological and histopathological findings definitively confirmed the diagnosis of tibial tuberculous osteitis.

The patient was started on a 9-month anti-tuberculosis regimen tailored for bone involvement. The intensive phase included a daily four-drug combination of isoniazid (5 mg kg^−1^ day^−1^), rifampicin (10 mg kg^−1^ day^−1^), pyrazinamide (25 mg kg^−1^ day^−1^) and ethambutol (15–25 mg kg^−1^ day^−1^) for 2 months. This was followed by a continuation phase with isoniazid and rifampicin for seven additional months.

## Discussion

Tuberculosis remains a major public health concern, particularly in developing countries where it is endemic, including Morocco [4,5]. Among extrapulmonary forms, tuberculous osteitis is a well-recognized but relatively uncommon manifestation, often associated with spinal tuberculosis (Pott’s disease) or tuberculous arthritis [3]. It typically follows an indolent course with nonspecific symptoms, contributing to delayed diagnosis [6].

In endemic settings such as Morocco, diagnostic delays may be compounded by limited awareness of skeletal tuberculosis among clinicians, particularly when symptoms mimic more common pathologies like chronic osteomyelitis or bone tumours [7].

Conventional radiography may be normal during early stages or show nonspecific findings such as osteolysis, periosteal reactions, soft tissue opacities or even pathological fractures [8]. The classic lacunar ‘pseudocystic’ pattern of bone condensation described by Jungling in 1920 remains a reference in the radiological identification of tuberculous osteitis [9]. The presence of bone sequestra may further complicate the diagnosis by mimicking neoplastic lesions [3].

Cross-sectional imaging, particularly CT, is valuable for assessing cortical destruction and the extent of soft tissue involvement, while MRI is more sensitive for detecting early bone marrow changes and abscess formation.

The bacteriological diagnosis of skeletal tuberculosis is challenging due to the paucibacillary nature of bone lesions. Direct Ziehl–Neelsen staining often lacks sensitivity. In our case, although visualization of AFB was limited by suboptimal staining, microbiological and molecular testing provided strong evidence for diagnosis. In contrast, PCR-based molecular techniques offer excellent specificity and rapid detection of *M. tuberculosis* DNA, even in smear-negative cases [10]. In our case, all diagnostic modalities – including microscopy, culture, PCR (GeneXpert MTB/RIF^®^) and histology – returned positive results, providing a robust microbiological and pathological confirmation.

Differential diagnosis can be difficult in the absence of respiratory symptoms or typical pulmonary lesions, as bone tuberculosis may mimic malignancies both clinically and radiologically [11]. Bone biopsy remains the gold standard for diagnosis, allowing for histological identification of caseating granulomas and microbiological confirmation. However, in certain settings where biopsy is not feasible, other sample types such as synovial fluid or fine needle aspirates may be used, although their diagnostic yield is lower. Sensitivity varies from 20 to 80% depending on the technique, while PCR-based detection improves specificity.

Management of tuberculous osteitis requires a prolonged antitubercular treatment regimen, typically consisting of a 2-month intensive phase with isoniazid, rifampicin, pyrazinamide and ethambutol, followed by a 7-month continuation phase with isoniazid and rifampicin. The extended duration is necessary to ensure eradication of bacilli in bone tissue. Surgical intervention is generally reserved for complications such as abscess drainage, sequestrum removal or joint instability. Supportive orthopaedic measures, including immobilization, are recommended to reduce pain and prevent deformity [12].

With timely diagnosis and appropriate treatment, the prognosis of tuberculous osteitis is generally favourable. Early intervention is essential to prevent irreversible bone damage and functional impairment [13].

## Conclusion

Osteoarticular tuberculosis remains a diagnostic and therapeutic challenge, particularly in endemic regions such as Morocco. Its nonspecific and often insidious presentation requires a high index of suspicion and a comprehensive diagnostic strategy that integrates radiological, microbiological and histopathological findings. Early and accurate diagnosis, followed by prompt initiation of antitubercular therapy and selective surgical management when indicated, is critical for favourable outcomes. Effective management relies on close multidisciplinary collaboration between clinicians, radiologists, pathologists and microbiologists to ensure timely diagnosis and personalized care.

## Abbreviations

AFB: acid-fast bacilli
AFB: acid-fast bacilli
CSF: cerebrospinal fluid
CT: computed tomography
CT: computed tomography
HIV: human immunodeficiency virus
MDR-TB: multidrug-resistant tuberculosis
MRI: magnetic resonance imaging
MRI: magnetic resonance imaging
PCR: polymerase chain reaction
TB: tuberculosis
WHO: World Health Organization
